# Retraction notice for: “MicroRNA-221 promotes cell proliferation, migration, and differentiation by regulation of ZFPM2 in osteoblasts” [Braz J Med Biol Res (2018) 51(12): e7574]

**DOI:** 10.1590/1414-431X20207574retraction

**Published:** 2020-08-17

Xingguo Zheng^1^, Jinhua Dai^2^, Haijun Zhang^1^, and Zhibin Ge^1^

^1^Department of Orthopaedics, Ningbo No. 2 Hospital, Ningbo, China

^2^Department of Clinical Laboratory, Ningbo No. 2 Hospital, Ningbo, China

Correspondence: Zhibin Ge: <gezhibin315@126.com>

**Retraction for:** Braz J Med Biol Res | doi: http://10.1590/1414-431X20187574 | PMCID: PMC6207289 | PMID: 30365725

On April 15, 2020, the Brazilian Journal of Medical and Biological Research (BJMBR) received a request from the Corresponding author Zhibin Ge to withdraw this manuscript: “*In a subsequent experiment, we repeated the experiment in this manuscript. Unfortunately, we had different results than before. This led to inaccurate conclusions in the manuscript. In order to ensure the accuracy of the articles we have published, we must request retraction. All authors agree to this retraction*”.

As per consensus between the Editors-in-Chief of the BJMBR and the Authors, the article has been retracted.

